# Changes in knowledge and awareness for a community-based cancer screening educational program

**DOI:** 10.1186/s13690-023-01144-w

**Published:** 2023-07-12

**Authors:** Shenghui Wu, Patricia Chalela, Amelie G Ramirez

**Affiliations:** 1 Department of Public Health and Exercise Science, Beaver College of Health Sciences, Appalachian State University, Boone, NC 28607 USA; 2 Institute for Health Promotion Research, Department of Population Health Sciences, UT Health San Antonio, San Antonio, 78229 USA

**Keywords:** Cervical cancer, Colorectal cancer, Breast cancer, Educational program, Health knowledge, Awareness, Hispanics, Screening, Health education

## Abstract

**Background:**

Cervical cancer (CC), colorectal cancer (CRC), and breast cancer (BC) are diseases that can be prevented/detected through early test. Through educational programs, individuals can become better informed about these cancers and understand the importance of screening and early detection. However, many people, especially low-income, low-educated, uninsured minority population groups, do not have their cancer screenings at the recommended intervals and do not receive appropriate and timely follow-up of abnormal screening results or timely treatment after diagnosis. A community-based educational program was developed to improve knowledge and awareness toward the screening of the three cancer types in a South Texas underserved population.

**Methods:**

Residents living in Laredo, Texas were invited to participate in the present educational program. From January 2020 to April 2021, participants were recruited using social media and flyer distributions in the general community. Participants received a free live web cancer education presentation delivered by bilingual community health educators, and online pre- and post-education surveys for CC, CRC, and BC separately. Pre-post changes in knowledge for individual items were compared using McNemar’s chi-squared tests.

**Results:**

Overall, the participants showed increases in CC (n = 237), CRC (n = 59), and BC (n = 56) screening knowledge and awareness after receiving the cancer screening education (*P*s < 0.05). After receiving the cancer screening education, 85–97% of participants had an intent to talk to a healthcare provider about CC/CRC/BC screening, 88–97% had an intent to get a CC/CRC/BC screening test in the next 12 months or at the next routine appointment, and 90–97% had an intent to talk about CC/CRC/BC with their family members or friends.

**Conclusions:**

A community-based educational program culturally and linguistically tailored help increase knowledge and awareness about cervical, colorectal, and breast cancer screening, and promote positive changes in population’s knowledge and awareness about the benefits of cancer screening. Future cancer screening educational programs in similar populations are warranted to reduce the risk of cervical, colorectal, and breast cancer.

**Table:** 

Text box 1. Contributions to the literature	
• Research has shown that many people, especially low-income, low-educated, uninsured minority populations, do not have their cancer screenings and do not receive appropriate and timely follow-up of abnormal screening results or timely treatment after diagnosis	
• We found that a community-based educational program can help increase knowledge and awareness about cervical, colorectal, and breast cancer screening, and promote positive changes in population’s knowledge and awareness about the benefits of cancer screening in underserved populations	
• These findings contribute to recognized gaps in the literature, i.e., developing and promoting cancer screening educational programs in underserved populations are warranted to reduce cancer risk	

## Background

Secondary prevention (such as screening) can be associated with reduced rates of incidence and mortality of cervical cancer (CC) [1–4], colorectal cancer (CRC) [4–6], and breast cancer (BC) [4]. CC, CRC, and BC continue to be the most frequently diagnosed cancers among Texas people, regardless of race and ethnicity, and they are the leading causes of cancer death [7], although the mortality rates for CRC and BC decreased while the rate for CC was not statistically significant increased [8] and the survival was improved for these cancers over time [9].

Detecting and treating CC, CRC, and BC at an early stage of the diseases, when treatment is more effective and treatment options and survival rates are greater, is an effective way to reduce morbidity and mortality from these diseases. There is strong evidence from randomized controlled trials and observational studies that cervical screening does offer protective benefits and is associated with a reduction in the incidence of invasive CC and CC mortality [3]. Screening is likely associated with reduction of CRC incidence after 10 years since baseline screening [5, 6]. Mammography screening reduces BC mortality and advanced cancer for women aged 50 years or older [10]. The screening tests may come with several risks, including the risk for false-positive and false-negative results as well as under- and overdiagnosis [11]. However, getting screening tests regularly may find CC, CRC, and BC early, when treatment is likely to work best [12].

However, many people, especially low-income, low-educated, uninsured minority population groups, do not have their cancer screenings at the recommended intervals and do not receive appropriate and timely follow-up of abnormal screening results or timely treatment after diagnosis[13]. Compared to the United States (U.S.) overall, residents in South Texas have lower per capita personal incomes ($31,965 vs. $54,446); higher rates of unemployment (5.3% vs. 3.7%), poverty (32.4% vs. 13.1%), and lack of insurance (29.6% vs. 8.9%); lower educational attainment (less than high school: 25.6% vs. 10.2%); and higher prevalence of obesity (30% vs. 23%) based on the latest available data [14–18]. Each of these factors may uniquely impact both cancer incidence and survival rates. Laredo, Texas is the fourth-most populous city in South Texas and third-most populated on the Mexico-United States border [15]. Based on the latest statistics from the U.S. Census Bureau, 30.8% poverty level of the population in Laredo live in poverty, much higher than the 15.5% poverty level of individuals in the U.S [19]. The latest all cancer age-adjusted incidence is 328 per 100,000 (49.5 for BC, 10.3 for CC, and 34.9 for CRC) and 3,297 all cancer survivors exist in Laredo [7]. The border region of South Texas is a medically underserved area, such as inadequate access to care (lack of health insurance and shortage of health care providers) [15, 20, 21] and cancer research is limited [20–23]. Therefore, the aim of our study is to first develop a community-based educational program to improve knowledge and awareness toward the screening of the three cancer types in the Laredo underserved population.

## Methods

Residents living in Laredo, Texas were invited to participate in the community educational program. From January 2020 to April 2021, 352 participants were recruited using media advertisements (University of Texas Health San Antonio Laredo Campus and Mays Cancer Center Facebook and Instagram), and flyer distributions throughout local public libraries, school districts, community centers, hospitals/clinics, universities/colleges in general community. The program contact information such as the telephone number and email address were listed on flyers. This project was exempt from an Institutional Review Board review as this was an educational program and no identifiable information was collected.

The educational materials were obtained from the American Cancer Society [24], Centers for Disease Control and Prevention [25], and Surveillance, Epidemiology, and End Results[26]. The educational materials include what is the cancer (CC/CRC/BC), mortality and early detection, causes and risk factors, and screening and treatment for each cancer type. Bilingual PowerPoint presentations and infographics were developed by the Institute for Health Promotion Research at University of Texas Health San Antonio. General topics for each cancer type included: What is cancer, general facts on incidence, mortality and survivorship, risk factors, warning signs, screening guidelines, treatment options, things to do to reduce cancer risks, and points to remember. Bilingual and bicultural community health workers were trained to promote and deliver the education program. The training included providing community health workers with education presentations and relevant materials, presentation practices, recruitment strategies (communication and coordination), education and intervention procedures, survey administration, and educational program delivery platform use.

Both English and Spanish survey questionnaires included deidentified-demographic information [such as age, sex, race (white, black or African American, American Indian or Alaska Native, Asian, and Native Hawaiian or Other Pacific Islander)/ethnicity (Hispanic or non-Hispanic), marital status, education, occupation, annual household income, and health insurance], ever had a CC/CRC/BC cancer screening, pre- and post-education questions (such as risk factors, symptoms, prevention, and early detection and screening), and post-education questions (such as intentions to get screening in the next 12 months or talk about BC/CC/CRC with family members or friends) for each cancer type based on the educational materials.

The educational program was delivered via a zoom platform by two health educators due to the COVID-19 pandemic. Health educators scheduled English and Spanish sessions for each cancer type separately. Participants needed to register for participation so as to receive a zoom meeting link, a survey link, and a unique ID number prior to each education session via emails/messages. Pre- and post-education data were collected using REDCap [27, 28]. REDCap is a secure, web-based software platform designed to support data capture for research studies, providing (1) an intuitive interface for validated data capture; (2) audit trails for tracking data manipulation and export procedures; (3) automated export procedures for seamless data downloads to common statistical packages, and (4) procedures for data integration and interoperability with external sources. Participants completed a pre-survey before the education presentation and completed a post-survey after receiving the education presentation using a RedCap survey link. Each session was around 60 min in duration. A total of 56 education sessions were delivered to participants.

### Statistical analyses

The education program sample was characterized with descriptive statistics. Pre-post changes in knowledge for individual items were categorical variables and compared using McNemar’s chi-squared tests. We dichotomized intent items (an intent to talk to a healthcare provider about cervical cancer screening, an intent to get a cervical cancer screening test, and an intent to talk about the cervical cancer with family members or friends) as “agree” or “disagree” responses. We calculated the proportions who changed their knowledge and awareness before and after education screening program. The relationships between socio-demographic factors and intent items for cervical cancer (the sample size did not allow for other cancer types) were examined using logistic regression models controlling for other covariates. Analyses were conducted using SAS (9.4, SAS Institute Inc., Cary, NC).

## Results

### Cervical cancer

Of the 237 participants, 182 (77%) completed both pre- and pos-surveys. The mean age of the participants was 38 [standard deviation (SD): 14.4] years (Table 1). Most of the participants (94%) were Hispanic, and white (96%), and had annual household income less than $50,000 (74%). Almost half of the participants had high school education or less (45%), had no insurance (50%), and were employed (47%), married (41%) or single (38%). Among these participants, 72% ever had a Pap smear, and 53% had a Pap smear during the last 2 years.

**Table 1:** Characteristics of the participants: community-based cancer screening educational program, Laredo, Texas, 2020–2021

Characteristics	Cervical Cancer (n = 237)			Colorectal Cancer (n = 59)			Breast Cancer (n = 56)	
	Number	Proportion (%)		Number	Proportion (%)		Number	Proportion (%)
Age (years)	37.65 ± 14.4*			42.96 ± 9.57*			38.87 ± 14.5*	
< 20	54	24.22		7	12.73		3	6.00
20–29	53	23.77		4	7.27		15	30.00
30–39	36	16.14		15	27.27		10	20.00
40–49	41	18.39		22	40		16	32.00
50–59	27	12.11		4	7.27		4	8.00
60–69	7	3.14		3	5.45		2	4.00
70–79	5	2.24						
Sex								
Men				3	5.56			
Women	237	100		51	94.44		56	100
Race								
White	195	96.06		53	98.15		51	96.2
Other	8	3.95		1	1.85		2	3.8
Birthplace								
United States	128	59.53		33	61.11		35	64.81
Other	87	40.47		21	38.89		19	35.19
Hispanic or Latino origin								
No	13	6.10		1	1.85		4	7.41
Yes	200	93.9		53	98.14		50	92.59
Marital status								
Married or living as married	91	41.36		33	61.11		25	46.3
Divorced	26	11.82		10	18.52		10	18.52
Widowed	5	2.27					3	5.56
Separated	14	6.36		2	3.7		1	1.85
Single, never been married	84	38.18		9	16.67		15	27.78
Education level								
High school or less	98	44.54		16	29.64		13	24.07
Above high school	122	55.45		41	75.94		41	75.92
Occupational status								
Employed full-time or part-time	104	47.06		41	75.93		28	51.86
Other	117	52.93		13	24.07		26	47.3
Annual household income								
$0-$49,999	144	73.45		34	62.96		33	61.11
$50,000 or more	47	23.97		20	37.04		21	38.89
Health insurance or health care plan								
None	107	49.54		14	25.93		22	41.51
Employer or others	109	50.46		40	74.08		31	58.5
Preferred Language								
English	77	46.95		33	55.93		39	69.64
Spanish	87	53.05		26	44.07		17	30.36
Ever had a Pap smear								
Yes	154	71.96						
No	60	28.04						
Had a Pap smear during the last 2 years								
Yes	112	52.58						
No	101	47.42						
After completing the session								
An intent to talk to a healthcare provider about cervical cancer screening								
Agree	171	93.96						
Disagree	11	6.05						
An intent to get a Pap smear and/or HPV test in the next 12 months or at the next routine appointment								
Agree	164	92.65						
Disagree	13	7.34						
An intent to talk about cervical cancer with family members or friends								
Agree	157	90.23						
Disagree	17	9.77						
Ever screened for colorectal cancer								
Yes				17	32.08			
No				36	67.92			
Methods screened for colorectal cancer								
FOBT/FIT (an at-home stool test)				10	55.56			
Colonoscopy				7	38.89			
Other				1	5.56			
An intent to talk to a healthcare provider about colorectal cancer screening								
Agree				31	96.88			
Disagree				1	3.13			
An intent to get screened for colorectal cancer in the next 12 months or at the next screening appointment								
Agree				28	87.51			
Disagree				4	12.51			
An intent to talk about colorectal cancer with family members or friends								
Agree				31	96.88			
Disagree				1	3.13			
Ever had a mammogram								
Yes							27	50
No							27	50
Had a mammogram during the last 2 years								
Yes							21	38.89
No							33	61.11
An intent to talk to a healthcare provider about breast cancer screening								
Agree							31	93.94
Disagree							2	6.06
An intent to get a mammogram in the next 12 months or at the next routine appointment								
Agree							31	93.94
Disagree							2	6.06
An intent to talk about breast cancer with family members or friends								
Agree							32	96.97
Disagree							1	3.03

* mean ± standard deviation

More women knew that the human papilloma virus (HPV) (85.3% for pre-survey vs. 94.9% for post-survey), smoking (42.2% vs. 69.6%), long term use of oral birth control pills (85.3% vs. 94.9%), diet low in fruits and vegetables (26.2% vs. 55.7%), overweight (32% vs. 57%) are risk factors of cervical cancer comparing post-survey to pre-survey (all *P*s < 0.05), and so is family history of cervical cancer (81% vs. 86%) although the difference was not statistically significant. More women knew that persistent pelvic/abdominal pain (78.7% vs. 87.1%), and vaginal bleeding after going through menopause (60% vs. 93%) are symptoms of cervical cancer (all *P*s < 0.05). Regarding the early detection of CC, more women showed increased knowledge comparing post-survey to pre-survey, although the differences for some items were not statistically significant: cervical cancer screening is not recommended only for women who have symptoms (83.9% for pre-survey vs. 85.1% for post-survey, *P* = 0.67); women should have a Pap test every 3 years at ages 21–29 and every 5 years at ages 30+ (32.5% vs. 36.3%, *P* = 0.20); and the Pap test together with the HPV test should be performed every 5 years starting at age 30 (64% vs. 75%; *P* = 0.04) (Table 2).

**Table 2:** Knowledge and awareness on cervical cancer screening before and after screening education delivery: community-based cancer screening educational program, Laredo, Texas, 2020–2021

	Presurvey		Postsurvey		*P*
	Number	Proportion (%)	Number	Proportion (%)	
**Risk factors of cervical cancer** (yes vs. no)					
Chances of developing cervical cancer are higher if there is a family history of cervical cancer	131	80.86	140	86.42	0.09
The human papilloma virus (HPV) is one of the major risk factors for cervical cancer	133	85.26	148	94.87	**0.003**
Smoking	100	42.19	165	69.62	**< 0.0001**
Long term use of oral birth control pills	80	33.76	125	52.74	**< 0.0001**
A diet low in fruits and vegetables	62	26.16	132	55.70	**< 0.0001**
Being overweight	75	31.65	135	56.96	**< 0.0001**
**Symptoms of cervical cancer** (yes vs. no).					
Persistent pelvic/abdominal pain	122	78.71	135	87.10	**0.02**
Unusual vaginal discharge that may contain blood	108	72.48	111	74.50	0.61
Vaginal bleeding after going through menopause	94	59.87	146	92.99	**< 0.0001**
**Prevention of cervical cancer** (yes vs. no).					
Using protection during sexual activity can help lower my chances of getting HPV	91	58.71	138	89.03	**< 0.0001**
Having an HPV infection means you will get cervical cancer (no vs. yes).	52	34.21	69	45.39	**0.03**
Cervical cancer can be prevented with regular screening	139	89.68	155	96.77	**0.008**
**Early detection of cervical cancer**					
Cervical cancer screening is recommended only for women who have symptoms (no vs. yes).	135	83.85	137	85.09	0.67
Should have a Pap test every 3 years at ages 21–29 (yes vs. no)	77	32.49	86	36.29	0.20
The Pap test together with the HPV test should be performed every 5 years starting at age 30 (yes vs. no).	103	66.03	117	75.00	**0.04**

After receiving cervical cancer screening education, 94% of the participants had an intent to talk to a healthcare provider about cervical cancer screening, 93% had an intent to get a Pap smear and/or HPV test in the next 12 months or at the next routine appointment, and 90% had an intent to talk about cervical cancer with their family members or friends (Table 1). We also observed a positive relationship between age and an intent to get a Pap smear and/or HPV test (odds ratio = 1.12; 95% CI 1.01, 1.23; *P* = 0.03), between annual household income and an intent to get a Pap smear and/or HPV test (odds ratio = 10.47; 95% CI 1.17, 94.08; *P* = 0.04), and between age and an intent to talk about cervical cancer with family members or friends (odds ratio = 1.14; 95% CI 1.04, 1.24; *P* = 0.004). For each year increase in age, there was an estimated 12% increase in the odds of an intent to get a Pap smear and/or HPV test. Compared with annual household income ≥$50,000, annual household income <$50,000 was associated with an estimated 17% increase in the odds of an intent to get a Pap smear and/or HPV test. For each year increase in age there was an estimated 14% increase in the odds of an intent to talk about cervical cancer with their family members or friends. Insurance status, education, and occupation were adjusted.

### Colorectal cancer

Of the 59 participants, 32 (54.2%) completed both pre- and pos-surveys. The mean age of the participants was 43 (SD: 9.6) years (Table 1). Most of the participants (94%) were female (94%), Hispanic (98%), and white (98%). Among these participants, 30% had high school education or less; 61% were married; 17% were single; 76% were employed; 63% had annual household income less than $50,000; 26% had no insurance; and 32% ever had screened for colorectal cancer.

More women knew that lack of physical activity (40.7% for pre-survey vs. 49.2% for post-survey, *P* = 0.31), heavy alcohol consumption (47.5% vs. 49.2%, *P* = 0.84), overweight/obesity (32.2% vs. 47.5%, *P* = 0.02), low fiber and high-fat diet (27.1% vs. 50.9%, *P* = 0.02) are risk factors of CRC comparing post-survey to pre-survey. Most items for other CRC screening knowledge and awareness were improved after education, although differences for some items were not statistically significant. Regarding the early detection of CRC, more women showed statistically significant increased knowledge comparing post-survey to pre-survey: a colonoscopy should be performed every 10 years starting at age 50 (44% for pre-survey vs. 88% for post-survey); a stool (poop) test [fecal immunochemical test (FIT) or fecal occult blood test (FOBT)] should be done every year starting at age 50 (50% vs. 81%) (Table 3).

**Table 3:** Knowledge and awareness on colorectal cancer screening before and after screening education delivery: community-based cancer screening educational program, Laredo, Texas, 2020–2021

	Presurvey		Postsurvey		*P*
	Number	Proportion (%)	Number	Proportion (%)	
**Risk factors of colorectal cancer**					
Lack of physical activity	24	40.68	29	49.15	0.31
Heavy alcohol consumption	28	47.46	29	49.15	0.84
Overweight/obesity	19	32.20	28	47.46	**0.02**
Low fiber and high-fat diet	16	27.12	30	50.85	**0.004**
Only people with family history of colon cancer will get the disease (no vs. yes)	30	93.75	31	96.88	1.00
A diet high in red meats and processed meats (lunch meat, hot dogs) increases chances of developing colorectal cancer (yes vs. no)	31	96.88	31	96.88	1.00
People younger than 50 years don’t get colon cancer (no vs. yes)	31	96.88	31	96.88	1.00
**Symptoms of colorectal cancer** (yes vs. no)					
Having blood in the stool or dark stools	31	96.88	30	93.75	1.00
Having a decrease in appetite	27	84.38	28	87.50	1.00
Experiencing unintentional weight loss	27	84.38	28	87.50	1.00
Even if I have no symptoms, I may still have colorectal cancer	29	90.63	29	90.63	1.00
**Early detection of colorectal cancer** (yes vs. no)					
In general, a colonoscopy should be performed every 10 years starting at age 50	14	43.75	28	87.50	**0.006**
A stool-based test (FIT/FOBT) checks your stool (poop) for blood	26	81.25	25	78.13	0.75
It is ok to skip colorectal cancer screening if do not have any symptoms (no vs. yes)	25	78.13	30	93.75	0.18
In general, a stool (poop) test (FIT or FOBT) should be done every year starting at age 50	16	50	26	81.25	**0.004**

After completing the colorectal cancer screening education, 97% of the participants had an intent to talk to a healthcare provider about CRC screening, 88% had an intent to get screened for CRC in the next 12 months or at her next routine appointment, and 97% had an intent to talk about CRC with their family members or friends (Table 1).

### Breast cancer

Of the 56 participants, 34 (61%) completed both pre- and pos-surveys. The mean age of the participants was 39 (SD: 14.5) years (Table 1). Most of the participants were Hispanic (93%) and white (96%). Among these participants, 25% had high school education or less; 46% were married; 28% were single; 52% were employed; 59% had annual household income less than $50,000; 42% had no insurance; and 50% ever had a mammogram.

More women knew that getting older (82.4% for pre-survey vs.88.3% post-survey), smoking (88.2% vs. 94.1%), drinking alcohol (76.5% vs. 91.2%) are risk factors of BC comparing post-survey to pre-survey, although the differences were not statistically significant. Regarding the early detection of BC, more women showed increased knowledge comparing post-survey to pre-survey. Most items for other BC screening knowledge and awareness were improved after education, although the differences were not statistically significant or were not able to be calculated as 100% of the participants provided correct answers after education: mammogram is not a blood test for breast cancer (94% for pre-survey vs. 100% for post-survey); women need to have not only once mammogram (97% vs. 91%); women should start getting mammograms at age 40 years (38% vs. 50%); and mammography or mammogram is the best way to detect breast cancer (94% vs. 100%) (Table 4).

**Table 4:** Knowledge and awareness on breast cancer screening before and after screening education delivery: community-based cancer screening educational program, Laredo, Texas, 2020–2021

	Presurvey		Postsurvey		*P*
	Number	Proportion (%)	Number	Proportion (%)	
**Risk factors of breast cancer**					
Getting older is a risk factor for breast cancer (yes vs. no)	28	82.4	30	88.3	0.63
Smoking increases my risk of breast cancer (yes vs. no)	30	88.2	32	94.1	0.69
Drinking alcohol increases my risk of breast cancer (yes vs. no)	26	76.5	31	91.2	0.23
Breastfeeding may decrease my risk of breast cancer (yes vs. no)	29	85.3	26	76.5	0.45
Having my mother or a sister with breast cancer means I am more likely-get it (yes vs. no)	33	97.1	32	94.1	1.00
Married women are more likely-get breast cancer (no vs. yes)	31	91.2	33	97.1	0.50
**Symptoms of breast cancer**					
A lump in the breast is always breast cancer (no vs. yes)	31	91.2	31	91.2	1.00
If a lump does not hurt, it is not breast cancer (no vs. yes)	30	88.2	33	97.1	0.38
A woman can have breast cancer and not have any signs or symptoms (yes vs. no)	33	97.1	32	94.1	1.00
**Early detection of breast cancer**					
A mammogram is a blood test for breast cancer (no vs. yes)	32	94.1	34	100	
If a woman had a mammogram once, she does not need to have a mammogram done again (no vs. yes)	31	91.2	33	97.1	0.63
What age should you start getting mammograms? (choice = 40 years)?	13	38.2	17	50.0	0.34
What is the best way-detect breast cancer (choice = mammography or mammogram)?	32	94.1	34	100	

After receiving the BC screening education, 94% of the participants had an intent to talk to a healthcare provider about BC screening, 94% had an intent to get a mammogram in the next 12 months or at the next routine appointment, and 97% had an intent to talk about BC with their family members or friends (Table 1).

## Discussion

This program found that participants demonstrated increases in CC, CRC, and BC screening knowledge and awareness after receiving the cancer screening education (*P* values < 0.05). We also observed a positive relationship between age and an intent to get a Pap smear and/or HPV test, between annual household income and an intent to get a Pap smear and/or HPV test, and between age and an intent to talk about CC with family members or friends.

To our knowledge, this is the first program to deliver cancer screening education for CC, CRC, and BC to the community in Laredo, Texas. Only one small study reported that 92 participants from El Paso, Brownsville, and Laredo were lack of knowledge about CRC and CRC screening and suggested that strategies were needed to educate Hispanic residents of border communities about CRC and to motivate them to undergo CRC screening [20]. Indeed, after receiving our cancer screening education, not only participants increased their knowledge and awareness on cancer screening for decreasing risk of CC/CRC and increasing early detection rate for BC, but also 85–97% of participants had an intent to talk to a healthcare provider about CC/CRC/BC screening, 88–97% had an intent to get a CC/CRC/BC screening test in the next 12 months or at the next routine appointment, and 90–97% had an intent to talk about CC/CRC/BC with their family members or friends. Although most of items for knowledge and awareness for BC screening were improved after education, differences were not statistically significant. One reason is that the statistical significance tests for some items were not able to be calculated as 100% of the participants provided correct answers after education. Future studies with a larger sample size are warranted to provide further information.

Our finding of the positive association between higher annual household income (≥$50,000) and increased awareness to get a cervical cancer screening test compared with less income (<$50,000) was supported by the prior evidence [29]. Interventions for these subgroups need to be based on effective strategies that have been found to reach underserved women. Community-based education interventions and establishment of local screening centers have been found to be effective approaches in rural settings [30]. Our program also suggested that awareness to get a cervical cancer screening test increased with age. As most (83%) of the participants were younger than 49 years, this positive association was not generalizable to the women with a large proportion of older groups. Facilities available for residents of Laredo to screen for these cancers include Laredo Medical Center [31], Doctors Hospital of Laredo [32], Gateway Community Health Center, Inc [33], and Mercy Ministries of Laredo [34]. The Gateway Community Health Center, Inc [33, 35] can provide cancer care for low-income and uninsured patients and immigrants in Webb County.

Our program has limitations. First, due to the coronavirus disease 2019 (COVID-19) pandemic, we delivered the cancer screening education sessions virtually which may limit people without internet access or electronic devices or those unable to use the internet or electric devices to participate. However, 92% had at least one type of computer and 85% had a broadband internet subscription among all American households in 2018 [36]. Thus, this limitation may not affect the findings substantially. Second, the online platform might be related to the fact that 23-46% participants did not complete the post-survey. Third, the sample sizes for CRC and BC are smaller than CC, and so some association analyses such as logistic regression models are unable to be estimated. Further studies with larger sample size for these analyses are warranted.

The present education program also has some strengths. First, our program first delivered cancer screening education to the underserved South Texas population and collected precious data during the COVID-19 pandemic. Second, we comprehensively collected data on demographic information, cancer screening, and pre- and post-education questions (such as risk factors, symptoms, prevention, and early detection and screening), thus we provided a relatively complete data on cancer screening education program. Third, the most important thing is that participants increased their knowledge and awareness on cancer screening after receiving the cancer screening education and they had intents to get cancer screening tests and disseminate their knowledge to family members or friends. This is the purpose of our educational program, and it helps establish the basis to realize our final goal – decrease the incidence and mortality of these cancers.

## Conclusion

This community-based educational program can help increase knowledge and awareness about cervical, colorectal, and breast cancer screening, promote positive changes in population’s knowledge and awareness about the benefits of cancer screening. Such programs may play an important role in addressing health disparities and informing underserved populations about recommended screening tests. Future cancer screening educational programs in similar populations are warranted to reduce the risk of cervical, colorectal, and breast cancer.

## Data Availability

The datasets generated during and/or analyzed during the current program are not publicly available due to privacy.

## List of Abbreviations

BC: Breast cancer
CC: Cervical cancer
COVID-19: Coronavirus disease 2019
CRC: Colorectal cancer
FIT: Fecal immunochemical test
FOBT: Fecal occult blood test
HPV: Human papilloma virus
SD: Standard deviation
U.S.: United States
